# Synergistic Effect of *Serratia fonticola* and *Pseudomonas koreensis* on Mitigating Salt Stress in *Cucumis sativus* L.

**DOI:** 10.3390/cimb47030194

**Published:** 2025-03-15

**Authors:** Sajid Ali, Murtaza Khan, Yong-Sun Moon

**Affiliations:** 1 Department of Horticulture and Life Science, Yeungnam University, Gyeongsan 38541, Republic of Korea; 2 Agriculture and Life Sciences Research Institute, Kangwon National University, Chuncheon 24341, Republic of Korea

**Keywords:** abiotic stress, biofertilizers, cucumber, consortia, PGPR, salinity, stress-related gene

## Abstract

Beneficial microbes enhance plant growth and development, even under stressful conditions. *Serratia fonticola* (S1T1) and *Pseudomonas koreensis* (S4T10) are two multi-trait plant growth-promoting rhizobacteria (PGPRs) that are resistant to saline conditions. This study evaluated the synergistic effect of these PGPRs on mitigating salinity stress (200 mM) in *Cucumis sativus*. Presently, the synergistic effect of both strains enhances the plant growth-promoting attributes of cucumber, and the growth parameters were significantly higher than those of uninoculated plants. The PGPR-treated plants revealed a significantly higher biomass and improved chlorophyll content. The inoculation of S1T1 and S4T10 and the synergistic effect of both promoted 23, 24, and 28% increases, respectively, in the fresh biomass and 16, 19.8, and 24% increases, respectively, in the dry biomass. Similarly, S1T1 and S4T10 and their synergistic effects led to 16.5, 28.4, and 38% increases, respectively, in the water potential and 18, 22, and 28% decreases, respectively, in abscisic acid (ABA). A reduction in the electrolytic leakage (EL) was additional proof of successful PGPR activities. Similarly, a decrease in the antioxidant levels, including those of malondialdehyde (21–30%), hydrogen peroxide (19–38%), and superoxide anions (24–34%), was observed, alongside an increase in antioxidant enzymes such as catalase (22–29%) and superoxide dismutase (17–27%). Additionally, the synergistic inoculation of the PGPRs enhanced the NaCl stress tolerance by upregulating the expression of the ion transporter genes *HKT1* (1–2-fold), *NHX* (1–3-fold), and *SOS1* (2–4-fold). Conclusively, the synergistic effect of the multi-trait PGPRs significantly enhances *C. sativus* L. growth under salt stress.

## 1. Introduction

Abiotic stress factors, such as salinity, drought, extreme temperature, heavy metals, and waterlogging, have become more frequent and severe because of global climate change, resulting in reduced crop growth and productivity [1,2]. According to estimates, abiotic stress is responsible for the loss of more than 50% of agricultural yields, and salt stress alone results in a 1–2% annual loss in arable land [3,4]. Salinity is a major problem in agricultural soil because of its adverse impact on different crops worldwide [1,5]. The increase in NaCl ions (Na^+^ and Cl^−^) in the soil causes salinity, where various factors including irrigation with saline water, the use of chemical fertilizers, and high evaporation contribute to salt accumulation and negatively impact different crop plants [6]. Salt stress can trigger several different unwanted responses in plants, lead to metabolic toxicity and reactive oxygen species formation, reduce photosynthesis, and affect the nutrient uptake capability of plants [7]. Moreover, an increase in the salt content causes ionic stress conditions that constrain plants from absorbing adequate amounts of nutrients and water from within close vicinity of the plant’s rhizosphere [8]. In other words, salt stress is a combination of osmotic, ionic, and oxidative stresses that lowers the water potential of leaves and tissues and creates ionic imbalance and osmotic stress [9].

Environmentally friendly strategies, such as applying plant growth-promoting rhizobacteria (PGPRs), are essential for tackling such stressful conditions and enhancing crop yields in the future [2]. Various direct and indirect processes have shown that PGPRs develop symbiotic relationships with plants and support plant growth under stressful conditions [10]. In nature, plant and soil bacteria continue to interact closely, where the bacteria are typically mutualistic and beneficial to plants. A plant root system produces exudates that significantly affect the growth of rhizospheric bacteria. Thus, the interactions between rhizobacteria and plants have been studied extensively [11], and communication between interacting partners involves physiological and molecular mechanisms, even under abiotic stress conditions. Rhizobacteria relieve stressful conditions using different mechanisms; some are known as direct mechanisms, while others are indirect mechanisms [10]. The functional roles of a plethora of multi-trait PGPR strains have been reported in crop nutrition and health, while the mitigation of abiotic stress conditions has been reported from various environmental conditions. Subsequently, some PGPR strains belonging to *Arthrobacter*, *Azobacetr*, *Azospirillium*, *Bacillus*, *Burkholderia*, *Enterobacter*, *Flavobacterium*, and *Pseudomonas putida* are used as inoculants to improve plant growth attributes under such stressful conditions [12,13,14].

The studies of Yuan et al. [15] and Gholizadeh et al. [16] reported that *HKT1*, *NHX*, and *SOS1* transporters work together to regulate ion balance and enhance salinity tolerance in plants by reducing Na⁺ toxicity while maintaining K⁺ levels. *HKT1* retrieves Na⁺ from the xylem, limiting its accumulation in aerial tissues. *NHX* transporters, located on the vacuolar membrane, sequester Na⁺ into vacuoles, mitigating cytoplasmic toxicity and preserving osmotic stability. *SOS1*, a plasma membrane Na⁺/H⁺ antiporter, expels Na⁺ from root cells into the rhizosphere to prevent excess accumulation, with its function regulated by calcium-binding proteins [15,16]. The use and efficacy of a single PGPR with one or more than one plant growth-promoting characteristics have been reported in different crops. However, the synergistic effect of PGPRs with multiple traits has received less attention. Using numerous PGPRs rather than a single bacteria in producing biofertilizers may be preferred because it synergistically affects nutrient mobilization and boosts efficacy, stability, and uniformity when utilized in different crops [14]. Our previous study reported on the multi-trait plant growth-promoting activities of both strains (*S. fonticola* (S1T1) and *P. koreensis* (S4T10)) individually and deposited the sequences in the NCBI database (MZ612851 and MZ612855). The study also revealed different activities of *S. fonticola* (S1T1) and *P. koreensis* (S4T10) including siderophore production, phosphate solubilization, and the production of indole-acetic acid (IAA), and the aminocyclopropane-1-carboxylic acid (ACC) deaminase enzyme [17]. These PGPRs can be used as biofertilizers, and their mode of application in the rhizosphere and phyllosphere needs to be investigated further in different crop plants.

*Cucumis sativus* L., commonly known as cucumber, is a plant species in the Cucurbitaceae family. Additionally, *C. sativus* L. is widely cultivated and serves as a versatile ingredient in various everyday meals. In the Republic of Korea, cucumber fruit is a substantial part of everyday meals consumed as part of traditional Kimchi recipes [18]. Different forms of fertilizers (including chemicals) are used to enhance the quality of the soil in which they grow. However, the applications of chemicals contaminate the soil, decrease soil fertility, and adversely affect groundwater quality [19]. Conversely, applying biofertilizers improves plant health and sustains soil fertility and the environment [20]. This study evaluated the synergistic effects of newly isolated *S. fonticola* (S1T1) and *P. koreensis* (S4T10) on cucumber plants under normal and NaCl stress conditions. Parameters such as electrolyte leakage (EL), relative water content (RWC), antioxidant activity, and pattern of gene expression in the salt-related genes under both normal and NaCl stress conditions were investigated to determine the role of these PGPRs on salt stress.

## 2. Materials and Methods

### 2.1. PGPR Strain Cultures

The isolation, characterization, and identification of *S. fonticola* (S1T1) and *P. koreensis* (S4T10) from plant species in Pohang Beach have previously been reported [17]. The cultures of these PGPRs were kept at 4 °C in about equal proportion of nutrient broth and glycerol (40%) for long-term storage. PGPR strains were added to the nutrient-rich Luria–Bertani (LB) broth medium (MB cell, Seoul, South Korea). This medium offers vital elements necessary for bacterial growth, including vitamins, minerals, and amino acids. The strains were cultured in the broth at 28 °C for 24 h to prepare the inoculum. 

### 2.2. Cucumber Seed Sterilization

The detailed procedure described by Ofek et al. [21] was used to sterilize the surfaces of the cucumber seeds. Briefly, the seeds were surface-sterilized by soaking and gentle shaking in 3% sodium hypochlorite for 90 s (Chungnam, Republic of Korea). The seeds were then placed into 70% ethanol (Ulsan, South Korea) for 90 s before being cleaned with sterile deionized water [21]. The sterile filter paper was used to dry the seed surface. 

### 2.3. Evaluation of the Range of Cucumber Seedling Tolerance to Salinity Stress

Surface-sterilized seeds were sown for germination in a 50-cell seed planting tray (one seedling per pot) containing 25 g of sterilized horticultural soil. Eight days later, uniform-sized seedlings were selected for transplantation. The selected seedlings were transplanted into 10 cm diameter pots (one seedling per pot) containing 210 g of soil and cultivated in a growth chamber in a natural dark and light photoperiod at 20 °C ± 2 °C with 55% relative humidity. The seedlings were watered to saturation thrice weekly; ten were considered per treatment. Different NaCl concentrations (0 mM, 100 mM, 200 mM, 300 mM, and 400 mM) were used to evaluate the range of plant tolerance to NaCl stress. 

### 2.4. Salt Stress Application, PGPR Interactions, and NaCl Stress

The experimental design used eight distinct sets of plants to assess selected PGPRs as inoculum under NaCl stress. The treatments were as follows: (i) control (plants without PGPRs and salt stress); (ii) S1T1-treated; (iii) S4T10-treated; (iv) both S1T1- and S4T10-treated; (v) salt-stressed only; (vi) S1T1 and NaCl stress; (vii) S4T10 and NaCl stress; (viii) S1T1 + S4T10 + NaCl stress. Autoclaved soil (210 g) was removed from each pot (12 × 10 cm), and the seedlings were grown for three consecutive weeks in a greenhouse (28.2 °C); seedlings were watered using either distilled water or NaCl solution (DUKSAN, Ansan, Republic of Korea). Previously, the NaCl tolerance-related growth of the PGPRs was examined in LB medium at various NaCl concentrations (0, 1, 2, and 3%), and growth was measured at OD_600_ (Supplementary Figure S1). The selected PGPRs were used for a subsequent experiment to assess any influence on the cucumber plants. According to the recommended protocols, 40 mL of the bacterial isolate (10^8^ CFU/mL) was applied twice to the autoclaved soil [22]. The bacterial suspension was applied twice before and after transplantation, with an interval of 48 h between applications. Subsequently, the plants were grown in the greenhouse at 28 ± 2 °C for five weeks, and salt stress was applied from week one to week five. A salt (NaCl) solution was used for high salt stress (200 mM), and three replicates of each treatment were prepared. Cucumber seedlings were subjected to high salt stress for one week to evaluate the effects of salinity on growth and development. Specifically, a 200 mM NaCl solution was utilized since this concentration is known to lead to significant osmotic and ionic stresses in plants [23,24]. The experiment focused on five-week-old seedlings since this represents a crucial developmental stage, during which the root systems and leaf structure are established. The seedlings were closely monitored for changes in various growth parameters, including plant height, overall biomass, and total chlorophyll content, and evaluated by using a SPAD meter (SPAD-502-Minolta Ltd, Osaka, Japan). When plants were harvested, shoot and root lengths and fresh and dried weights were measured. The dry weights following all treatments were calculated after the plants were dried in an oven at 70 °C for 48 h.

### 2.5. Determination of Leaf Relative Water Content (LRWC) 

For the evaluation of LRWC, the Barrs and Weatherley [25] method was adopted with a few minor modifications, including the imbibition duration (24 h), to calculate the leaf water potential percentage (%). Identical leaf varieties were removed from the plants in each treatment and weighed (leaf fresh weight, LFW). To calculate the turgid fresh weight of leaves (TFWL) after calculating the LFW, in a sealed container, the leaves were submerged in the distilled water and left to float for 24 h. Then, leaves were blotted dry using blotting paper before being weighed. The leaves were heated to 70 °C in an oven for 24 h to achieve the dry biomass of the leaf (DWL). Subsequently, to determine the LRWC, the values of the LFW, TW, and DWL were applied to the following formula (Equation (1)):

(1) $$LRWC(\%)=(\frac{LFW-DWL}{TFWL-DWL})\;\times \;100$$

### 2.6. Measuring the Electrolyte Leakage (EL)

The protocols reported by Dellagi et al. [26] were adopted to measure the electrolyte leakage with a few minor modifications. Here, a total of 200 g of fresh leaves samples was used in this study. The samples, each measuring 5 mm in length, were placed in test tubes containing 10 mL of distilled deionized water to assess the extent of EL. All tubes were sealed in a water bath heated up to 32 °C. An electrical conductivity meter was used to measure the initial electrical conductivity of the medium (EC1) (HURIBA Twin Cond B-173, Japan) after 120 min. The final electrical conductivity (EC2) of the sample was measured after autoclaving at 121 °C for 20 min and cooling to 25 °C. The quantity of electrolyte leakage was calculated using the following formula (Equation (2)):

(2) $$Percentage\;\left(\%\right)E.L=(\frac{EC1}{EC2})\;\times \;100$$

### 2.7. Evaluation of Oxidative Stress Markers

Antioxidant activity analysis was conducted by pulverizing 400 mg of freeze-dried leaf tissues using a cold mortar and pestle before homogenizing the tissue in phosphate buffer (50 mM, pH 7.5; DUKSAN, Ansan, Republic of Korea) with polyvinylpyrrolidone (PVP) 1.0% (*w*/*v*), EDTA (0.1 mM), and Triton X-100 0.5% (*w*/*v*) [27]. Superoxide anions (SOAs) were precisely measured at a wavelength of 580 nm using the previously described method [28,29]. Likewise, the H_2_O_2_ levels were measured following various treatments using the previously described methods [30]. Lipid peroxidation (LPO) values were determined at a wavelength of 532 nm using the comprehensive method reported by Srivastava et al. [31].

### 2.8. Enzymatic Antioxidant Activity

The catalase (CAT) activity was evaluated using the methodology outlined by Radhakrishnan and Lee [32], and the resulting absorbance was measured at 240 nm. Spectrophotometer measurements were taken at 560 nm using the detailed technique for determining superoxide dismutase (SOD) activities [33].

### 2.9. Estimation of Endogenous Abscisic Acid (ABA)

The endogenous ABA content was extracted and measured using a previously described detailed approach [34]. The extracts were dried, methylated with diazomethane, and analyzed using GC/MS-SIM (6890N GC and 5973 mass selective detector, Agilent Technologies Inc. Palo Alto, CA, USA). Me-ABA and Me-[2H6]-ABA were quantified based on ion responses (m/e 162, 190 and m/e 166, 194, respectively) using Lab-Base (ThermoQuest, Manchester, UK) data system software. 

### 2.10. Gene Expression Analysis Using qRT-PCR

Gene expression was analyzed through quantitative real-time PCR (qRT-PCR) following a previously described method [35]. RNA was extracted from treated cucumber leaf samples using TRIzol solution (Invitrogen, Carlsbad, CA, USA). Reverse transcription was carried out using the DiaStar™ RT kit (SolGent, Daejeon, Republic of Korea) in an Eco™ real-time PCR thermocycler. The qRT-PCR reactions included cDNA, 2X Real-Time PCR Master Mix with SYBR Green I (BioFACT™, Daejeon, Republic of Korea), and gene-specific primers (Table 1). β-actin was used as a reference gene for normalization, and expression levels in control plants were used to assess the impact of treatments on gene expression. 

### 2.11. Statistical Analysis 

All data were collected in triplicate to ensure reliability and statistical robustness. To analyze the differences among treatment means, Duncan’s Multiple Range Test (DMRT) was conducted using SAS version 9.2 software, which allows for multiple pairwise comparisons while controlling Type I error rates. The statistical analysis facilitated the identification of significant variations among treatments. Following the statistical evaluation, the results were visualized using GraphPad Prism software (Version 6.01, San Diego, CA, USA), which enabled the creation of high-quality graphical representations for better interpretation and comparison of the data.

## 3. Results

### 3.1. Effect of PGPRs (S. fonticola and P. koreensis) on Plant Growth Under Salt Stress

**Figure 1:**
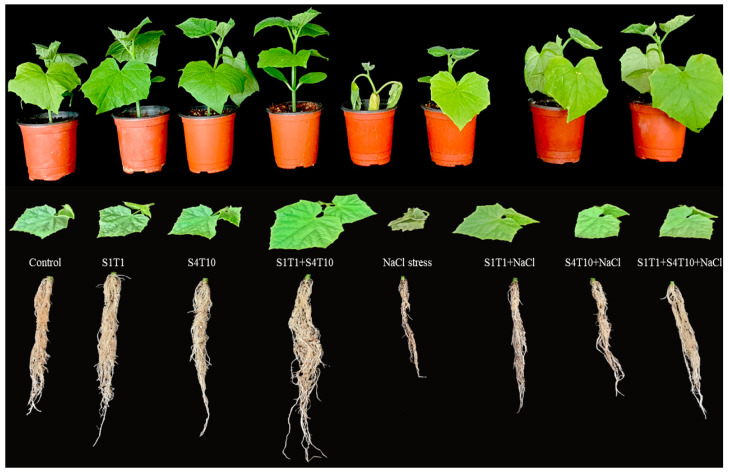
Effects of PGPR (*S. fonticola* (S1T1) and *P. koreensis* (S4T10)) inoculation on cucumber plants under NaCl stress. The figure presents the phenotypic response of cucumber plants subjected to different treatments, including control (untreated), inoculation with *S. fonticola* (S1T1), *P. koreensis* (S4T10), and their combined application (S1T1+S4T10), both in the absence and presence of NaCl-induced salinity stress.

NaCl stress adversely affected the growth and development of cucumber plants; however, this effect was alleviated following inoculation with multi-trait PGPRs. Plants inoculated with PGPRs demonstrated improved growth compared to the uninoculated control plants (Figure 1). 

The inoculation of the PGPRs revealed a prominent effect on the growth of the cucumber plants and significantly mitigated the harmful effects of salinity stress. The results showed that the root and shoot lengths of the cucumber plants were greatly improved by applying PGPRs compared to the untreated control plants. In contrast to S1T1, the rhizobacterium *P. koreensis* (S4T10) treatment significantly promoted plant growth during NaCl-induced stress (Table 2). The inoculation with PGPRs such as S1T1 and S4T10 demonstrated prominent effects on the cucumber plants and significantly augmented the fresh biomass by 23.06% and 24.34%, respectively. By contrast, the synergistic effect of inoculating with both PGPRs revealed a 28.45% increase in the fresh biomass of the plants under normal control conditions. Similarly, the shoot length under *S. fonticola* (S1T1) and *P. koreensis* (S4T10) were 16.92% and 21.08% higher, respectively, compared to the control plants under normal conditions. In comparison, the consortia of *S. fonticola* (S1T1) and *P. koreensis* (S4T10) revealed a more than 20% increase in the shoot length of the inoculated plants under salinity conditions (Table 2).

The PGPR inoculations significantly alleviated the adverse effects of salt stress and enhanced the growth attributes of the cucumber plants. In comparison to the untreated control plants, those treated with *P. koreensis* (S4T10) and *S. fonticola* (S1T1) demonstrated a significant increase in the fresh and dry weight, shoot and root length, and chlorophyll content. This suggests that the application of these bacterial strains positively influenced plant growth-promoting attributes, including the chlorophyll content, potentially enhancing the photosynthetic efficiency and overall plant health. These results showed that the cucumber plants under salt stress had considerably smaller shoots (39.8%) and reduced fresh biomass (36.4%) than the control plants. Compared to the untreated control plants, the plants treated with *P. koreensis* (S4T10) and *S. fonticola* (S1T1) exhibited significantly higher chlorophyll contents. Moreover, the plants treated with *S. fonticola* (S1T1) and *P. koreensis* (S4T10) under NaCl stress had 24.56% and 25.52% higher chlorophyll contents, respectively. Thus, the combined application of S1T1 and S4T10 under salinity stress revealed improved plant chlorophyll contents. The treatment with *P. koreensis* (S4T10) and S1T1 also significantly improved the biomass (fresh and dry). The synergistic effect of inoculating with both PGPRs significantly enhanced the plant growth-promoting attributes under stressful conditions (Table 2; Figure 1). Furthermore, applying *P. koreensis* (S4T10) and *S. fonticola* (S1T1) significantly alleviated salt stress compared to in the untreated control plants.

### 3.2. PGPRs’ Role in Leaf Water Potential Under NaCl Stress

**Figure 2:**
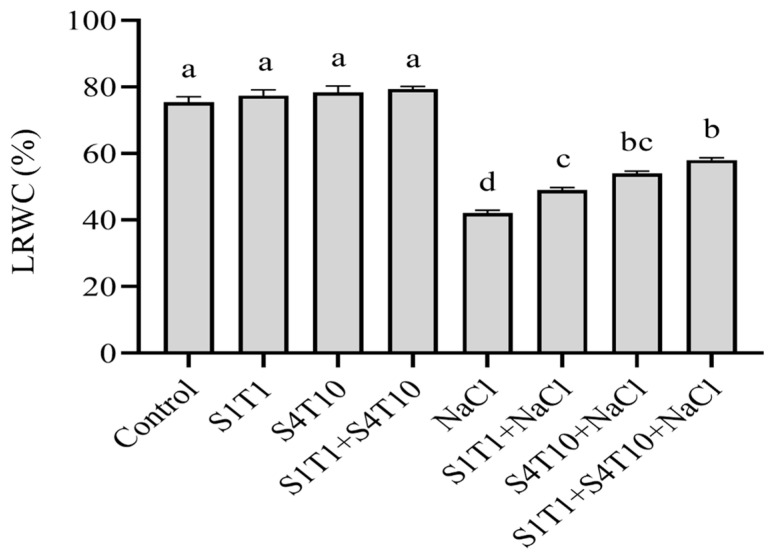
Influence of the PGPRs on the LRWC of cucumber plants under both normal and salt stress conditions. The data are presented as the mean values and standard error bars from three replicates. Different letters on the mean bars indicate significant differences according to DMRT analysis.

The capability and capacity of a plant to store water are significantly affected by salt stress. The non-inoculated control cucumber plants exhibited an inferior leaf water status to those treated with the PGPRs. The combined application of *P. koreensis* (S4T10) and *S. fonticola* (S1T1) promoted a significant increase in the LRWC under salinity conditions (Figure 2). Compared to the *P. koreensis* (S4T10) and *S. fonticola* (S1T1)-inoculated cucumber plants, the LRWC of the control plants was 12.6% and 11.4% lower, respectively. Conversely, NaCl stress had a more distinct effect on the uninoculated control plants. The uninoculated control plants had a considerably lower water potential than the *P. koreensis* (S4T10)- and *S. fonticola* (S1T1)-inoculated plants under salt stress. When *P. koreensis* (S4T10) and *S. fonticola* (S1T1) were applied synergistically to the cucumber plants under salt stress, the resulting leaf water potential was increased by 38.0%. In contrast, the *S. fonticola* (S1T1)- and *P. koreensis* (S4T10)-treated plants under NaCl stress revealed LRWCs of 16.5% and 28.4%, respectively.

### 3.3. Effects of PGPRs on Electrolyte Leakage, MDA, H_2_O_2_, and SOAs Under NaCl Stress

**Figure 3:**
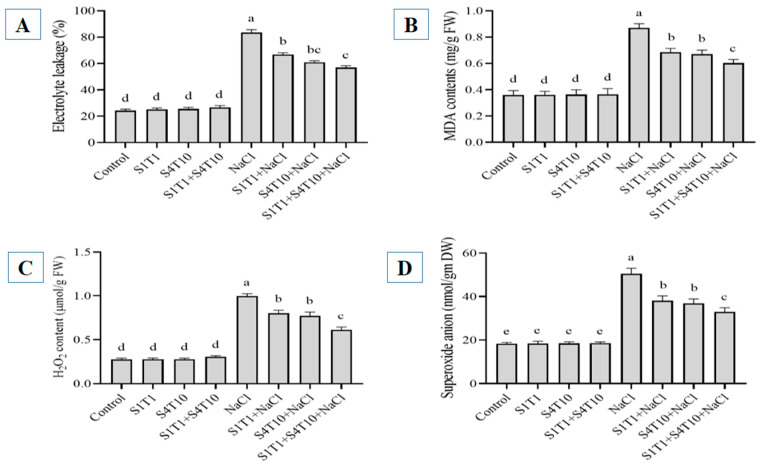
Effect of PGPRs on (**A**) leaf electrolyte leakage, (**B**) MDA levels, (**C**) H_2_O_2_ content, and (**D**) superoxide anion levels in cucumber plants under both normal and NaCl stress conditions. Data represent the mean ± standard error from three replicates. Different letters on the bars denote statistically significant differences based on DMRT analysis.

The EL, MDA, H_2_O_2_, and superoxide anion contents were assessed to determine the impact of the PGPRs and NaCl stress on the membrane integrity of the plant. The findings indicated a notable increase in electrolyte leakage, MDA, H_2_O_2_, and superoxide anion levels under NaCl-induced stress (Figure 3). The plants under salt stress revealed more electrolyte leakage from the leaf tissues than the control (treated only with water) plants. Comparatively, applying S1T1 and S4T10 individually and their combined application under NaCl stress reduced electrolyte leakage up to 19, 27, and 31%, respectively (Figure 3A). Salt stress adversely affected the membranes of these plants compared to the plants treated with PGPRs. The analysis of the MDA contents revealed the extent of lipid peroxidation (LPO); higher MDA levels were observed in the cucumber plants treated with NaCl compared to those in the normal control plants. In contrast, the individual and synergistic inoculation of the PGPRs decreased the MDA levels of the cucumber plants by 21, 23, and 30%, respectively, under salinity conditions (Figure 3B). 

Likewise, the ROS content was evaluated in the cucumber plants subjected to NaCl stress. Salt stress enhances the production of ROS, such as H_2_O_2_ and superoxide anions (SOAs). Moreover, the H_2_O_2_ level increased in response to NaCl stress. However, the H_2_O_2_ production in the cucumber plants was reduced significantly following the application of the PGPRs: 19% after the *S. fonticola* treatment and 22% following the *P. koreensis* treatment (Figure 3C). A similar pattern was observed in the superoxide anion (SOA) levels of the cucumber plants exposed to NaCl stress (Figure 3D). Indeed, SOA production was reduced significantly in the plants inoculated with *S.fonticola* (24%) and *P. koreensis* (27%) compared to the plants under NaCl stress conditions.

### 3.4. Effects of PGPRs on Antioxidant Enzymes Under NaCl Stress

**Figure 4:**
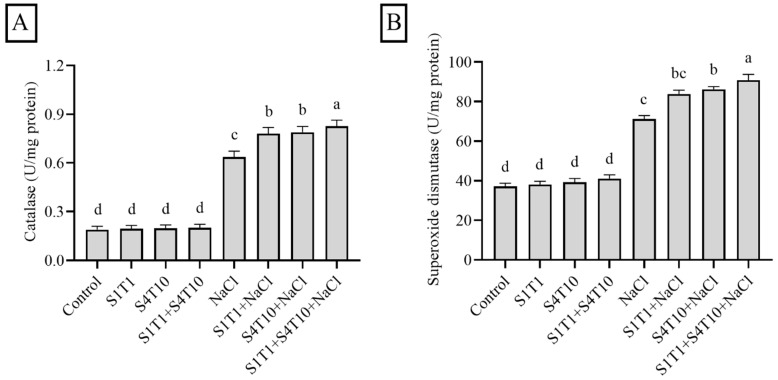
Effect of PGPRs on (**A**) catalase activity and (**B**) superoxide dismutase. The data are presented as the mean and standard error bars from three replicates. Different letters on the mean bars indicate significant differences according to DMRT analysis.

Plants activate antioxidants to regulate ROS generation and mitigate the detrimental effects of external stressors, including NaCl stress. Higher salt concentrations affect both soil properties and microbial processes, including respiration and enzyme activity. The enzymatic activity of SOD and CAT in the cucumber plants under normal and NaCl stress conditions, with and without PGPRs, was assessed (Figure 4). PGPR application under NaCl stress enhanced the SOD and CAT activity compared to that in the plants subjected to NaCl stress alone. The inoculation of *S. fonticola* (S1T1) and *P. koreensis* (S4T10) increased CAT biosynthesis by 22% and 23%, respectively. Furthermore, the combined application of the PGPRs further enhanced CAT production to 29% under salt stress conditions (Figure 4A). Likewise, the cucumber plants showed a similar pattern for SOD under NaCl stress conditions. Under salt stress conditions, the PGPRs enhanced SOD biosynthesis, whereby S1T1 produced a 17% increase and S4T10 produced an approximately 21% increase in the biosynthesis of SOD. On the other hand, the synergistic application of the PGPRs revealed a 27% increase in the activity of SOD (Figure 4B). Therefore, the levels of the analyzed antioxidant enzymes increased in the cucumber plants upon the inoculation with the multi-trait PGPRs.

### 3.5. Regulation of Endogenous ABA by PGPRs Under NaCl Stress

**Figure 5:**
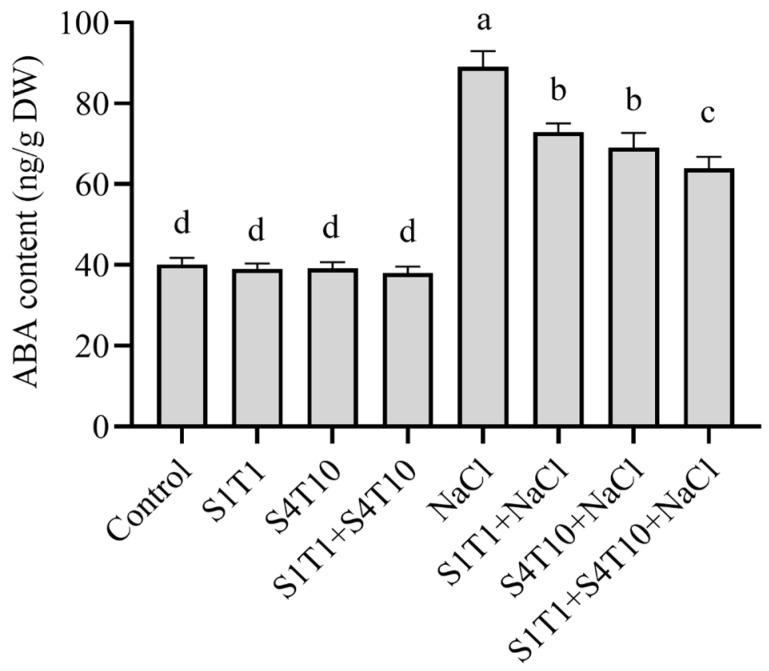
Effects of PGPRs on abscisic acid (ABA) regulation under NaCl stress. Different letters on the mean bars indicate significant differences according to DMRT analysis.

ABA is essential in modulating plant growth and performance, particularly in response to abiotic stressors, such as NaCl stress. Furthermore, PGPRs have been reported to promote prominent effects on regulating ABA in plants under stressful conditions. Therefore, the impact of PGPRs on the ABA content in the cucumber plants was assessed under NaCl stress conditions. Based on the research results, the plants under stress had significantly higher ABA concentrations than those in the control plants. Conversely, the plants treated with PGPRs under NaCl stress exhibited a modulated ABA content. As a result, under NaCl stress settings, the levels of ABA produced increased exponentially, in contrast to the plants treated with *S. fonticola* (S1T1) and *P. koreensis* (S4T10), which showed decreases of 18% and 22%, respectively. By contrast, the combined application of these PGPRs demonstrated a 28% decrease in the ABA content under salt stress conditions (Figure 5). 

### 3.6. Expression of NaCl Stress-Related Genes and Effects of PGPRs

Plants upregulate the expression of candidate genes under stressful conditions. Recent studies have reported that salt-responsive genes are modulated to combat the unwanted effects of salinity. Therefore, sodium transporters play a crucial role in plant defense. These transporters can be either antiporters or symporters; the former remove sodium ions from the root cell and redistribute them throughout the body to reduce the adverse effects of salt stress and restore water homeostasis. The expressions of three putative genes (*HKT1*, *SOS1*, and *NHX*) were examined to increase the systematic knowledge of the potential mechanism through which cucumber plants activate NaCl stress tolerance.

**Figure 6:**
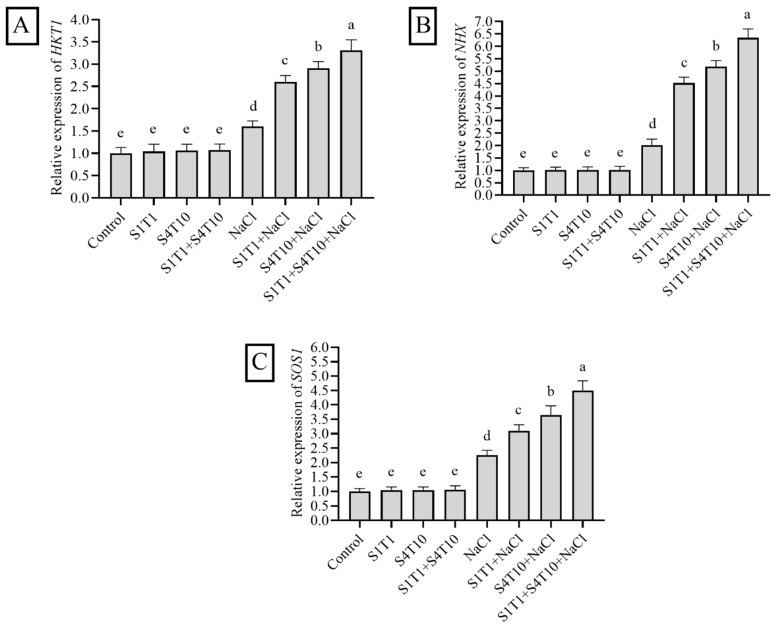
Effects of PGPRs on gene expression under normal and salt stress conditions. Leaf samples were collected from plants across all treatment groups to analyze gene expression. The relative expression levels of (**A**) HKT1, (**B**) NHX, and (**C**) SOS1 genes under normal and salt stress conditions were quantified using β-actin as the reference gene. Data are presented as mean ± standard error from three replicates, with different letters on the bars indicating statistically significant differences based on DMRT analysis.

*HKT1*, *NHX*, and SOS1 were upregulated in the cucumber plants under NaCl stress. On the other hand, transcript accumulation of the *HKT1*, *NHX*, and SOS1 genes was augmented in the cucumber plants inoculated with PGPRs. The expression of *HKT1* was enhanced by 1-fold under *S. fonticola* (S1T1) and 1.3-fold under *P. koreensis* (S4T10) compared to that in the NaCl-stressed control plants. Meanwhile, the combined application of S1T1 and S4T10 promoted a 1.7-fold increase in the expression of *HKT1* (Figure 6A). Thus, applying *S. fonticola* (S1T1) and *P. koreensis* (S4T10) to cucumber plants under NaCl stress can induce adaptability, which results in a significant increase in *HKT1*.

Similarly, the *NHX* gene expression increased significantly by 0.9-fold, 1.4-fold, and 2.3-fold upon the inoculation with S1T1 and S4T10 and their synergistic application, respectively, compared to the salt stress control plants (Figure 6B). The same pattern was observed for the *SOS1* gene, whereby a significant increase in expression was observed in the cucumber plants treated with the PGPRs (Figure 6C). The application *S. fonticola* (S1T1) and *P. koreensis* (S4T10) and their synergistic effect on cucumber plants improved NaCl resistance by increasing *SOS1* expression by 2.5-fold, 3.17-fold, and 4.34-fold, respectively, compared to in the uninoculated cucumber plants under salt stress (Figure 6C).

## 4. Discussion

Major environmental stress factors adversely affecting plant development and agricultural productivity include salinity, drought, heavy metals, extreme temperature, and ultraviolet (UV) radiation [36]. Among these factors, salt stress alone affects approximately 11% of the world’s irrigated land, which can result in a loss of more than 50% of crop yields in species sensitive to salt [4,37]. Salinity stress inhibits plant growth by increasing sodium uptake, increasing the amount of endogenous ABA, generating increased and toxic amounts of ROS, and decreasing the photosynthetic rate and potassium uptake [38]. Thus, salt stress has detrimental effects on the morphological, biochemical, and molecular aspects of plants.

The use of PGPRs can have significant environmental impacts, particularly on soil health and microbial diversity. PGPRs enhance soil fertility by improving the nutrient availability, increasing organic matter decomposition, and promoting plant–microbe interactions. They contribute to soil structure stabilization and increase water retention, reducing erosion risks [39]. Microbial inoculation offers a sustainable and cost-effective alternative to chemical amendments, genetic modifications, and advanced irrigation techniques, offering significant ecological benefits. Unlike chemical fertilizers, which contribute to soil degradation and environmental pollution, microbial inoculants enhance nutrient cycling, improve soil structure, and promote long-term fertility while minimizing synthetic input dependency [40]. Compared to genetic modifications, which require extensive regulatory approval and pose potential ecological risks, microbial inoculation naturally enhances plant resilience without altering genetic integrity. Additionally, while advanced irrigation strategies optimize water use, microbial inoculants improve drought tolerance by enhancing root development and soil water retention, thereby reducing irrigation demands. This approach supports sustainable agricultural practices by maintaining soil biodiversity, minimizing environmental impacts, and ensuring long-term productivity [40,41]. 

Several approaches have been used to mitigate the adverse impacts of abiotic stressors, including salt stress, and to enhance crop plant growth and productivity [20], including using various chemicals, producing genetically modified crops, and modern agricultural methods and irrigation systems. Ultimately, the current agricultural system and crop production greatly benefit from the contributions of these approaches. However, natural ecosystems have consequently suffered, since most fertilizers and chemicals end up in fields where they are wasted or kill beneficial microbes. Notably, various microbial plant biostimulants have recently been used to improve crop yields, enhance abiotic stress tolerance, and improve nutrient utilization [42]. The findings in this paper offer insight into improving the approach of applying PGPRs to combat NaCl stress. The rhizobacteria *S. fonticola* and *P. koreensis* have synergistic efficiency in stimulating plant growth-promoting features to induce salinity stress tolerance. 

In the current investigation, the cucumber plant growth and development were efficiently supported by the selected PGPRs (*S. fonticola* and *P. koreensis*), which also reduced the detrimental effects of NaCl stress. Nadeem et al. [43] also reported that PGPRs rescued cucumber plants following exposure to salt stress. Furthermore, Sapre et al. [44] reported that the application of PGPRs reduced the toxic effects of salt stress on pea plants, and the inoculation of rhizobacteria enhanced the parameters of pea seedling development under salinity stress. Furthermore, the inoculation of rhizobacteria decreased salt stress by altering physiological, biochemical, and molecular factors. In addition, the PGPR-inoculated plants displayed reduced electrolyte leakage and H_2_O_2_ concentrations under NaCl stress compared to those of the uninoculated seedlings. 

Salt stress leads to water deficit conditions in plant cells, affecting plant growth and development. The first observable sign of salt stress is a decrease in the RWC; hence, assessing the RWC is a good method for evaluating the water condition of the plant [45]. Under salt stress conditions, applying *S. fonticola* (S1T1) and *P. koreensis* (S4T10) enhanced the RWC compared to that of control plants. The current findings are consistent with those of Singh et al. [46], in that PGPRs assist the plants under salt stress. Abiotic stresses, including salt stress, induce electrolyte leakage and increase the MDA, H_2_O_2_, and superoxide anion contents [47]. Conversely, applying PGPRs reduces the production of these oxidative stress markers [18]. Similarly, salt concentrations that are too high interfere with microbial processes, including respiration and the activity of soil enzymes, and affect the physicochemical characteristics of the soil. PGPR application increases the activities of antioxidant enzymes, including CAT and SOD [48]. The findings of this study are also in line with those of these previous studies, and although salt stress increased the production of oxidative stress markers, the inoculation with *S. fonticola* and *P. koreensis*, particularly via the root zone, lowered their production. These multi-trait PGPRs significantly enhance cucumber growth and resilience under NaCl stress through several key mechanisms. Firstly, the PGPRs improve nutrient acquisition by solubilizing essential minerals, such as phosphorus, producing siderophores and ACC deaminase, thus supporting root development and overall growth in saline conditions [49]. Secondly, the PGPRs produce plant hormones, such as IAA, which promote root elongation and cell division, helping cucumber plants absorb more water and nutrients despite the inhibitory effects of salt [50,51].

ABA is an essential hormone that is crucial for growth and stress adaptation. Plants possess an improved ability to withstand abiotic stresses after activating physiological changes that improve survival and preserve water [52,53]. To reduce water loss during water shortage conditions, the plant can also control the opening and closing of stomata—microscopic pores on leaves. Furthermore, ABA affects cell division and elongation, which normally slows growth under stress to conserve resources. ABA also affects the development of roots, assisting them in adjusting to changes in the availability of nutrients and water. Abiotic stress conditions cause an increase in the levels of ABA in plants. Intriguingly, these results showed that ABA production was considerably reduced in the presence of the PGPRs compared to in the control plants under stress conditions. Although some studies suggested that adding bacteria to plant roots and leaves could boost the degree of ABA accumulation, the effect may vary depending on the microbe and plant species [54,55].

The coordinated action of transporters including *HKT1*, *NHX*, and *SOS1* enables plants to maintain ion homeostasis and improve their salinity tolerance by minimizing Na⁺ toxicity while preserving essential K⁺ levels. *HKT1*, *NHX*, and *SOS1* are key transporters involved in salinity stress tolerance by regulating Na⁺ exclusion, vacuolar compartmentalization, and ion homeostasis in plants. *HKT1* (typically, a mediator of relatively selective Na^+^ transport) functions as a Na⁺ transporter that facilitates Na⁺ retrieval from the xylem, preventing its excessive accumulation in aerial tissues. *NHX* transporters, primarily localized in the vacuolar membrane, mediate Na⁺ sequestration into vacuoles, reducing cytoplasmic Na⁺ toxicity and maintaining cellular osmotic balance. *SOS1*, a plasma membrane Na⁺/H⁺ antiporter, actively extrudes Na⁺ from root cells into the rhizosphere, preventing Na⁺ overload in plant tissues and modulation by calcium-binding proteins [15]. These transporters allow plants to maintain ion homeostasis and improve salt tolerance. Several PGPRs increase the ability of a plant to withstand salt stress [16,56]. By regulating ion transporter gene expression, PGPRs improve the resistance of plants to salt stress. Plants that grow in salty environments experience difficulties, such as ionic imbalances and elevated osmotic stress levels, which can hinder their development. By controlling the expression of particular ion transporter genes, which are critical for preserving ion homeostasis and controlling the absorption and distribution of vital nutrients, PGPRs help resolve these problems [16]. Moreover, PGPRs assist plants in more effectively managing excess salt by modifying the expression of these genes, ultimately reducing the negative effects on plants and maintaining development and yield in difficult environmental conditions. 

Salt stress tolerance in plants is conferred via various stress-related genes involved in signal transduction, ion transporters, transcription regulation, and metabolic pathways. The current study also found that applying *S. fonticola* (S1T1) and *P. koreensis* (S4T10) significantly increased the expression of *HKT1*, *NHX*, and *SOS1* in response to salt stress. Furthermore, the study by Liu et al. [57] reported that the inoculation of *Bacillus amyloliquefaciens* enhanced the expression of *HKT1* in the shoots of *Arabidopsis thaliana*. The enhancement in the growth attributes under salinity can be connected with the improved expression of the aforementioned genes under the inoculation of the PGPRs. Conclusively, applying multi-trait PGPRs could be an efficient way to confer NaCl stress resistance in crop plants. In the present study, the synergistic application of S1T1 and S4T10 mitigated NaCl (200 mM) stress in cucumber plants. The experimental data showed that the multi-trait PGPRs *S. fonticola* (S1T1) and *P. koreensis* (S4T10) played a pivotal role in the recovery of cucumber plants by activating antioxidants (SOD and CAT), modulating the phytohormone (ABA) levels, maintaining the RWC, and regulating ion transporter genes (*HKT1*, *NHX*, and *SOS1*). Therefore, the current study validates using a consortium of PGPRs to produce microbial plant biostimulants for the increased growth and quality yield of crop plants growing under saline stress conditions. Applying PGPRs in cucumber cultivation can promote growth and stress tolerance in saline areas, thereby encouraging sustainable agricultural methods. Moreover, future studies must be designed to include dose-dependent trials to evaluate the efficacy of PGPRs across different stress intensities, strain-specific quantification techniques, and confirm the survival and interaction dynamics of more than one strain in soil. 

## 5. Conclusions

Overall, *Cucumis sativus* L. yielded prominent benefits from synergistic inoculation with two multi-trait PGPRs, *S. fonticola* (S1T1) and *P. koreensis* (S4T10). This study demonstrates that combining these PGPRs increases plant stress tolerance and improves growth indices, including the biomass and chlorophyll content. The observed improvements in the fresh and dry biomass, water potential, and a decrease in ABA and electrolyte leakage demonstrate the efficiency of these PGPRs in reducing salinity stress. The ability of these PGPRs to increase plant resistance is further supported by the downregulation of oxidative stress indicators and the overexpression of ion transporter genes. Thus, the synergistic inoculation of these PGPRs appears to be a viable method for enhancing the growth and tolerance of plants under saline conditions. 

## Figures and Tables

**Table 1:** Primer sequences of *SOS1*, *HKT1*, and *NHX1*.

Symbol of Gene	Forward Primer	Reverse Primer
*SOS1*	5′-AGGAAGGTTCAAAGCCTAGTG-3′	5′-CATGAGTAAATGTGGGGTGCA-3′
*HKT1*	5′-GGACCTCTCCACCTTGTCGT-3′	5′-CTGCTACCGTTTGTTTGTCACTCT-3′
*NHX1*	5′-TGCTTTTGCCACCCTTTCA-3′	5′-TTCCAACCAGAACCAATCCC-3′

**Table 2:** Impact of NaCl stress on plant growth with and without *S. fonticola* (S1T1) and *P. koreensis* (S4T10). The data presented show the mean values from three replicates. Different letters indicate significant differences according to DMRT analysis.

Treatment	Shoot Fresh Weight (gm/plant)	Shoot Dry Weight (gm/plant)	Shoot Length/(cm)	Root Length/(cm)	Chlorophyll Content/(SPAD)
Control	23.35 ± 1.32 ^b^	5.98 ± 1.18 ^b^	22.41 ± 1.11 ^b^	21.27 ± 0.14 ^b^	40.11 ± 1.20 ^b^
S1T1-Treated	27.62 ± 0.24 ^a^	7.12 ± 0.35 ^a^	26.86 ± 0.23 ^a^	27.81 ± 1.81 ^a^	44.08 ± 1.05 ^a^
S4T10-Treated	28.15 ± 1.21 ^a^	7.41 ± 0.17 ^a^	26.90 ± 0.22 ^a^	27.08 ± 0.35 ^a^	44.62 ± 0.12 ^a^
S1T1+ S4T10 Consortia-Treated	29.17 ± 0.11 ^a^	7.60 ± 1.15 ^a^	28.21 ± 0.41 ^a^	30.67 ± 0.56 ^a^	44.98 ± 0.46 ^a^
NaCl (200mM) Stress	14.83 ± 1.41 ^e^	3.68 ± 0.22 ^e^	13.47 ± 1.12 ^e^	15.34 ± 0.41 ^e^	29.23 ± 0.22 ^e^
S1T1+ NaCl Stress	18.25 ± 0.64 ^d^	4.28 ± 0.56 ^d^	15.75 ± 0.32 ^d^	18.17 ± 0.11 ^d^	36.41 ± 0.48 ^d^
S4T10 + NaCl Stress	18.44 ± 0.52 ^d^	4.41 ± 0.47 ^d^	16.31 ± 0.65 ^d^	19.05 ± 0.52 ^d^	36.69 ± 0.34 ^d^
Consortia (S1T1+ S4T10) + NaCl Stress	19.05 ± 1.54 ^c^	4.58 ± 0.64 ^c^	17.04 ± 0.41 ^c^	20.13 ± 0.55 ^c^	37.05 ± 0.61 ^d^

## Data Availability

Datasets generated during the current study are available from the corresponding author on reasonable request.

## Supplementary Materials

The following supporting information can be downloaded at: https://www.mdpi.com/article/10.3390/cimb47030194/s1.
