# Supplementary figures and images for: Synergistic Effect of Serratia fonticola and Pseudomonas koreensis on Mitigating Salt Stress in Cucumis sativus L
Source: Curr Issues Mol Biol. 2025 Mar 15;47(3):194. doi: 10.3390/cimb47030194 (PMC11941737; doi:10.3390/cimb47030194)

**Supplementary Figure S1:** PGPR growth at different NaCl concentrations (0, 1, 2, and 3%)

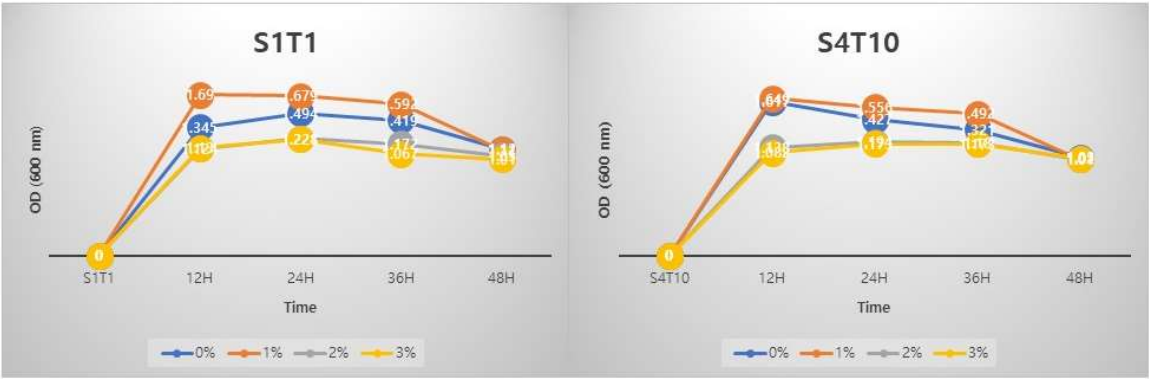

Supplement: Supplementary file 1 [file cimb-47-00194-s001.zip › cimb-3497556-supplementary.pdf]
